# A Biofeedback App to Instruct Abdominal Breathing (Breathing-Mentor): Pilot Experiment

**DOI:** 10.2196/13703

**Published:** 2019-09-30

**Authors:** Corinna Anna Faust-Christmann, Bertram Taetz, Gregor Zolynski, Tobias Zimmermann, Gabriele Bleser

**Affiliations:** 1 wearHEALTH Department of Computer Science Technische Universität Kaiserslautern Kaiserslautern Germany

**Keywords:** mobile health, relaxation, pain management, biofeedback, respiration, breathing exercises, feasibility study

## Abstract

**Background:**

Deep and slow abdominal breathing is an important skill for the management of stress and pain. However, despite multiple proofs on the effectiveness of biofeedback, most breathing apps remain limited to pacing specific breathing patterns, without sensor feedback on the actual breathing behavior.

**Objective:**

To fill this gap, an app named *Breathing-Mentor* was developed. This app combines effective visualization of the instruction with biofeedback on deep abdominal breathing, based on the mobile phone’s accelerometers. The aim of this pilot study was to investigate users’ feedback and breathing behavior during initial contact with the app.

**Methods:**

To reveal the possible effects of biofeedback, two versions of the mobile app were developed. Both contained the same visual instruction, but only the full version included additional biofeedback. In total, 40 untrained participants were randomly assigned to one of the two versions of the app. They had to follow the app’s instructions as closely as possible for 5 min.

**Results:**

The group with additional biofeedback showed an increased signal-to-noise ratio for instructed breathing frequency (0.1 Hz) compared with those using visual instruction without biofeedback (*F*_1,37_=4.18; *P*<.048). During this initial contact with the full version, self-reported relaxation effectivity was, however, lower than the group using visual instruction without biofeedback (*t*_37_=−2.36; *P*=.02), probably owing to increased cognitive workload to follow the instruction.

**Conclusions:**

This study supports the feasibility and usefulness of incorporating biofeedback in the Breathing-Mentor app to train abdominal breathing. Immediate effects on relaxation levels should, however, not be expected for untrained users.

## Introduction

### Mobile Stress Management

Chronic stress has been identified as a critical factor that influences people’s physical and mental well-being [1,2]. However, the effect of a stressor on an individual’s well-being also depends on his or her coping mechanism [3]. In addition to stress management group interventions and self-help literature, the use of stress management apps makes it possible nowadays to learn a broad range of problem-focused and emotion-focused coping methods [4-6]. Moreover, relaxation methods are also commonly integrated in apps for the management of chronic pain [7,8], chronic diseases [9], and anxiety [10]. Through interactive design and gamification, such apps can potentially increase the users’ motivation [11,12]. This could reduce the economic burden for the health care system [13]. There are first indications that some stress management apps are indeed effective [14,15], underpinning the usefulness of this prevention approach.

### Breathing Apps

Deep and slow diaphragmatic breathing can lead to a state of relaxation. Therefore, it is frequently taught as a basic strategy for the management of stress, anxiety, posttraumatic stress disorder [16], and pain [17]. Traditionally, breathing trainings are guided by health professionals, but the increasing importance for technology-driven approaches such as health apps can be attributed to financial reasons [13]. A broad range of apps specially designed for breathing trainings are available, but breathing exercises are also regularly incorporated in stress and anxiety management apps [5,10,18]. Most of these apps simply pace a distinctive breathing pattern, using audio or visual instructions. Regarding the effectiveness of these instructions, it has been shown that a wave-based visualization of the desired breathing pattern can be more easily followed compared with a circle-based visualization or a traditional audio instruction [19].

Besides pacing, providing biofeedback is another approach for breathing trainings (eg, [20-24]). With biofeedback, information from 1 or multiple sensors is used to gain greater awareness of physiological functions. Besides breathing rate, current stress management apps also target skin conductance [25] and heart rate [26,27]. Most mobile biofeedback solutions, however, require additional costly devices with integrated sensors (eg, a belt [28], wearable textile sensors [21,29], or clothing-adhered biosensors [30]).

In this study, biofeedback refers to feedback about the movement of the abdomen during a breathing task. One example for this kind of biofeedback is the BellyBio Interactive Breathing app for iOS devices by RelaxLine. It uses the mobile phone’s built-in accelerometers to capture the abdominal breathing movements. For deep and slow breathing, the sound of the ocean is transformed to relaxing music. However, so far, no study has analyzed the effectiveness of such abdominal breathing feedback. Moreover, the app is recommended only for people who are already familiar with breathing exercises. Direct instructions should be used for novices instead [19].

The *Breathing-Mentor* app is a biofeedback breathing app that was developed to provide such direct instructions. It combines the effective wave-base visualization of the desired breathing pattern [19] with biofeedback on the actual breathing behavior, using the mobile phone’s accelerometers. This approach allows comparing the desired breathing pattern with the actual breathing behavior in real time.

To investigate the feasibility and usefulness of the additional biofeedback, a control version without biofeedback, that is, with visual instruction only, was implemented as well. For this purpose, we conducted a user study to reveal how people who are unfamiliar with breathing exercises deal with both versions of *Breathing-Mentor*. The focus of this study was to determine the users’ ability to follow the breathing instructions and their subjective usage experience.

## Methods

### The Interface of Breathing-Mentor

### 

**Figure 1:**
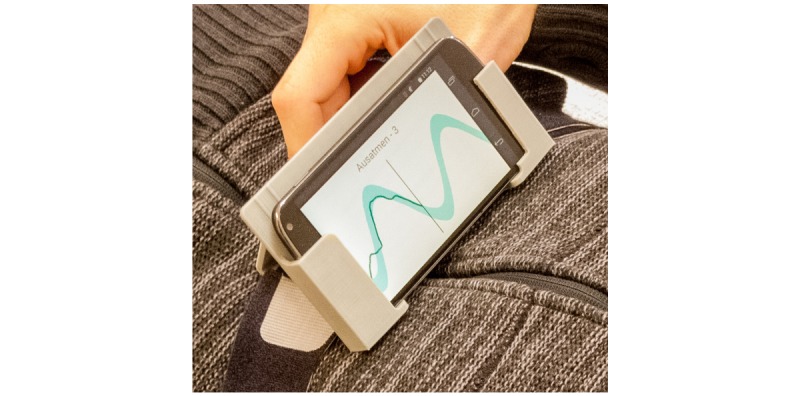
The Breathing-Mentor training user interface combines graphical (moving sine wave) and text instructions (inhale/exhale, counting from 1 to 4) for deep, slow abdominal breathing with biofeedback (dark line, not present in the control version).

The biofeedback signal is drawn over the sine wave (dark line, not present in the control version). It is obtained from the mobile phone’s accelerometers, given that the mobile phone is correctly positioned on the user. The latter is supported through an interactive calibration procedure. During the study, the mobile phone was fixed in a custom (three-dimensional [3D]-printed) frame, and the latter was fixed with an elastic band around the upper abdomen. Figure 1 shows the setup and the training user interface.

### Signal Processing Approach of Breathing-Mentor

The overall signal processing approach for transforming the accelerometer measurements into the breathing signal as visualized on the screen and used for data analysis is detailed below.

**Figure 2:**
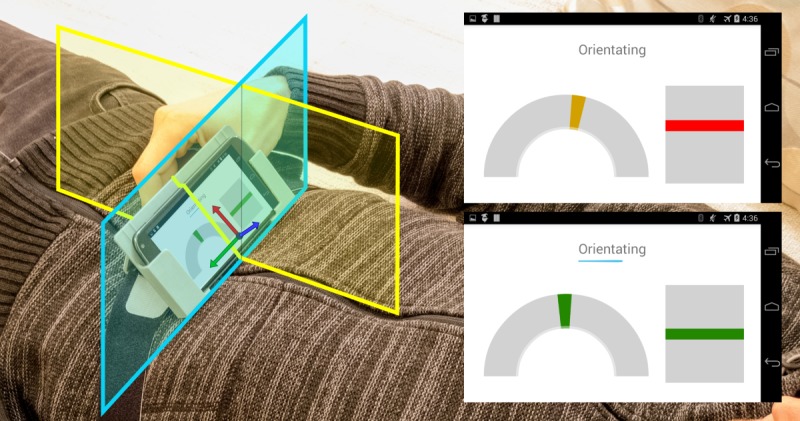
Positioning of the smartphone on the upper abdomen and interactive calibration procedure with traffic light feedback, aiding a repeatable positioning during the study. The yellow and cyan rectangles indicate the sagittal and transversal body planes, respectively. The coordinate system denotes the sensor coordinate frame, in which the accelerometer measurements are given. In the user interface, the half circle refers to the alignment in the smartphone’s xy-plane and the rectangle refers to the alignment in the xz-plane. For a successful alignment (through manually adjusting the position of the custom frame on the upper abdomen), both marks were required to be in the green area for five seconds.

Accelerometers measure 3D linear acceleration, a combination of body acceleration and acceleration resulting from gravity, in the local sensor coordinate system. As the participants are stationary during breathing training, the acceleration resulting from gravity constitutes the major portion of the measurement. Moreover, in the training target pose, this component provides information about lateral and anterior and posterior tilt of the mobile phone with regard to the sagittal and transversal body plane, respectively (see Figure 2). The basic idea is that—with the frame including the mobile phone being placed on the upper abdomen—abdominal breathing results not only in small up and down movements of the mobile phone but also in a change of the mobile phone’s orientation, where the tilt change relative to the transversal body plane is dominant. This again results in acceleration measurements with major dispersion direction approximately in the sagittal plane.

These assumptions were confirmed in pretests (1 min, 3 trials) with 5 persons already trained in abdominal breathing. For these trials, the frame including the mobile phone was positioned by the investigator (instructed by the algorithm developer) with its base above the center of the upper abdomen, so that the mobile phone’s long edge was approximately leveled, and the display was facing the person. The recorded accelerometer data from these trials were then used to obtain the major dispersion direction as the first principal component. A reference range (representing deep abdominal breathing) was extracted by projecting all recorded accelerometer vectors onto this principal component and calculating the average minimum and maximum values over the test persons.

### Calibration Procedure

The average accelerometer vector was also used as reference vector for aiding a repeatable positioning of the custom frame on the participants of the study and thus improving the validity and reliability of the extracted breathing signal. For this, the app provided an interactive calibration procedure with traffic light feedback on the angle deviations between the currently measured accelerometer vector and the reference vector in the xy-plane and in the xz-plane (green: <5°, orange: <15°, red: otherwise; see Figure 2). The angle deviations were calculated using scalar products between the respective vectors. This is based on the assumption that the mobile phone is kept rather stationary during the procedure, and therefore, the accelerometer measures mainly acceleration resulting from gravity, as mentioned above. The angle deviations in the xy-plane and in the xz-plane can be controlled by slowly moving the custom frame including the mobile phone on the upper abdomen laterally or forward and backward, respectively. For a successful alignment, the deviation was required to be in the green area (below 5°) in both planes for 5 seconds. The breathing signal was then obtained from the live accelerometer measurements by applying an infinite impulse response filter (resistor-capacitor low-pass filter) with cut-off frequency of 0.5 Hz, projecting the filtered measurements on the major dispersion direction again using the scalar product and scaling the result so that the reference range mapped to (−1, 1) according to the target sine wave.

### Pilot Study

To investigate the feasibility and usefulness of the additional biofeedback from the user perspective, we conducted a user study to reveal how people who are unfamiliar with breathing exercises deal with *Breathing-Mentor* compared with those using the control version of the app without additional biofeedback.

#### Study Protocol

The study was conducted in accordance with the Declaration of Helsinki, and the protocol was approved by the local Ethics Committee of the Department for Social Sciences. All participants (see Participants section) gave their informed consent for inclusion in the beginning. They were randomly assigned to the experimental group (EG) with biofeedback or the control group (CG) without biofeedback. In the beginning, previous experience with breathing exercises for relaxation was screened as described by Chittaro and Sioni [19]; see the Results section for details. Then, the investigator fixed the custom frame including the mobile phone (LG Nexus 4, sensor: InvenSense MPU-6050) over the participant’s clothes on the upper abdomen using the elastic band while ensuring that the clothes did not fall in folds. The calibration procedure was then performed to ensure correct positioning of the mobile phone for all participants for this study (see Calibration Procedure section for details). Participants lay horizontally during the whole procedure with their head placed comfortably on a pillow. This allowed a direct view on the mobile phone’s display.

The 3 measurement blocks are described in Table 1. Each block lasted 5 min, with breathing performance being recorded with the mobile phone’s accelerometers (see Signal Processing Approach of Breathing-Mentor section). The baseline block with no mobile phone-guided instruction was included to check if there were systematic differences of abdominal breathing patterns between the 2 groups. In the training block, participants were asked to follow the instructions given by the app as closely as possible, while breathing with the abdomen. The objective was to find out whether the required abdominal breathing pattern (6 cycles/min, 0.1 Hz) could be followed more easily with additional biofeedback on the breathing performance. Although deep abdominal breathing normally increases relaxation sensation in experienced users of breathing exercises, this is not necessarily the case for people who are unfamiliar with breathing tasks. Therefore, a questionnaire regarding the app’s effectiveness to support the breathing exercise and its effectiveness to evoke relaxation were assessed directly after the training block (questionnaire provided by Chittaro and Sioni [19]; see the Results section for details). The postmeasurement block was identical to the baseline block without mobile phone-guided instructions. It was included to test whether a single 5-min training session is already enough to evoke changes in the abdominal breathing patterns without further training.

**Table 1:** Description of the 3 measurement blocks.

Block sequence	Verbal instruction	Screen content	Control questions directly after the block
Baseline	Please breathe as slowly and deeply as possible with the abdomen.	Blank screen. The word *start* appears for 5 seconds. The word *stop* appears after 5 min.	The instruction was easy to follow (1=totally disagree—5=totally agree)
Training	Please follow the instructions on the screen as closely as possible while breathing with the abdomen.	Interface of Breathing-Mentor (see Figure 1), the dark line for biofeedback was not included for the control group.	Questionnaire on the app’s effectiveness (1=totally disagree—5=totally agree)
Post	Please breathe as slowly and deeply as possible with the abdomen.	Blank screen. The word *start* appears for 5 seconds. The word *stop* appears after 5 min.	The instruction was easy to follow (1=totally disagree—5=totally agree)

#### Participants

A total of 40 participants took part in the pilot study. One person from the CG was excluded owing to a chronic respiratory disease, resulting in a final sample size of 39. The mean age was 26.51 years (range 20-42 years, SD 4.41 years). Groups did not differ significantly with regard to age (*t*_37_=−0.93, *P*=.36) or sex ratio (males/females=9/10 in the CG, 10/10 in the EG, χ^2^_1_=0.03, *P*=.87).

#### Statistical Analyses for the Baseline

As no special breathing frequency was instructed in the baseline block, the power of all slow breathing–related frequency bands (0.055-0.195 Hz, width 0.01 Hz each) was compared between both groups in a variance analysis with repeated measurements to check for systematic differences between the groups. Neither systematic group effects nor interaction of group with frequency bands was expected for the baseline block.

#### Statistical Analyses for the Training Block

For the training block, the following 2 measures of the objective breathing behavior comparable with the study of Chittaro and Sioni [19] were calculated for each minute of the 5-min interval:

The first measure, the spectral power in the recommended frequency band (0.09-0.11 Hz), indicates how intensely the respiratory act is performed for the recommended frequency band. The second measure, respiratory signal-to-noise ratio (SNR), describes the ratio between the power of the recommended breathing frequency band (0.09-0.11 Hz) and the power in the entire breathing spectrum (excluding the band of the recommended frequency, the 0-0.05 Hz band to remove low-frequency fluctuations, and the direct current offset; see [19] for details). It reflects the ability of participants to correctly follow the instructions provided by the app.

Both, the spectral power in the recommended frequency band as well as the respiratory SNR are expected to increase in both groups for the training block owing to the visual instruction for the 0.1-Hz breathing rate, compared with the baseline condition. If the additional biofeedback actually enhances performance during the breathing exercise, there should be a main effect of group for both dependent measures. The additional within-subject factor *time* (5 steps, 1 min each) allows investigating changes in performance over time. Both groups are expected to increase performance over time for both dependent measures.

#### Statistical Analyses for the Postmeasurement Block

To test whether a single 5-min training session is already enough to cause changes in the abdominal breathing patterns toward the requested breathing frequency (0.1 Hz), the spectral power in the recommended frequency and the SNR of the postmeasurement block were compared in both groups with the baseline in 2 variance analyses with repeated measures.

#### Statistical Methods

For single comparisons, *t* tests for independent samples (group comparisons) and *t* tests for dependent samples (comparisons between blocks and minutes) are described. Please note that the given sample size only allows to reveal large effect sizes (0.8). *F* and *P* values are described in the context of variance analyses and *t* and *P* values for *t* tests.

## Results

### Comparability of Groups Before Training

The screening for previous experience with breathing exercises for relaxation revealed no systematic differences between groups (see Table 2 for details). Although most participants were aware that there is a difference between abdominal and thoracic breathing and that the former can be used for relaxing purposes, only few participants actually practiced breathing and meditation exercises in their daily lives.

**Figure 3:**
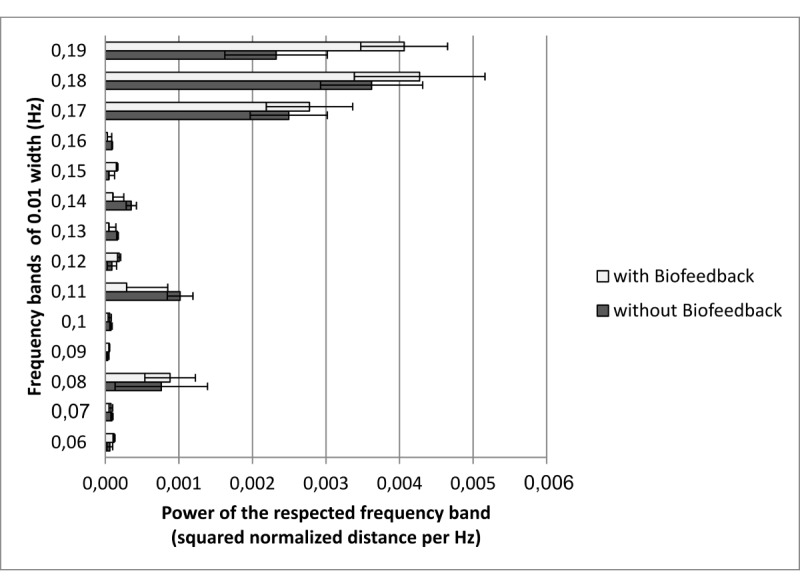
Mean powers of frequency bands in the baseline block for both groups. Error bars indicate standard error of the mean.

For the baseline block, there was a main effect of frequency bands with more power for frequency bands near the normal breathing rate (0.2 Hz; see Figure 3 for details). There were no main effect of group and no interaction between group and frequency bands (see Table 3 for details). Summarizing, the baseline measurements did not reveal any systematic group differences for objective slow abdominal breathing behavior. The subjective ratings on how easy the instruction was to follow did not reveal group differences either (*t*_37_=0.56, *P*=.58; EG: mean 4.55, SD 0.69; CG: mean 4.68, SD 0.82).

**Table 2:** Screening of previous experience with breathing exercises for the control group and experimental group. Absolute frequency of yes and no answers, chi-square values, and *P* values are described for each item.

Item	Control group (yes/no)	Experimental group (yes/no)	Chi-square (*df*)	*P* value
Do you know the difference between abdominal and thoracic breathing?	15/4	16/4	0.01 (1)	.94
Do you know that abdominal breathing is used in the context of relaxing exercises?	13/6	13/7	0.05 (1)	.82
Do you use breathing exercises for relaxation?	4/15	5/15	0.08 (1)	.77
Do you meditate regularly? (at least once a month)	2/17	4/16	0.67 (1)	.41

**Table 3:** Results of the analysis of variance for the power of frequency bands in the baseline block.

Factor	*F* (*df*)	*P* value
Frequency bands	32.21 (13,481)	<.001
Group	0.36 (1,37)	.56
Frequency bands × group	1.36 (13,481)	.18

### Effects of Additional Biofeedback

In both groups, there was an increase of the spectral power in the recommended frequency (CG: *t*_18_=3.47, *P*=.003; EG: *t*_19_=6.12, *P*<.001) as well as in the SNR (CG: *t*_18_=5.88, *P*<.001; EG: *t*_19_=4.16, *P*=.001) in the training block compared with the baseline block (see Table 4 for means and standard deviations).

To reveal changes over time, 5 time blocks of 1 min each were included as repeated measurements variable to investigate group differences in breathing performance.

For the spectral power in the recommended frequency, the analysis of variance revealed neither significant main effects nor an interaction between group and time (see Table 5 for details).

**Figure 4:**
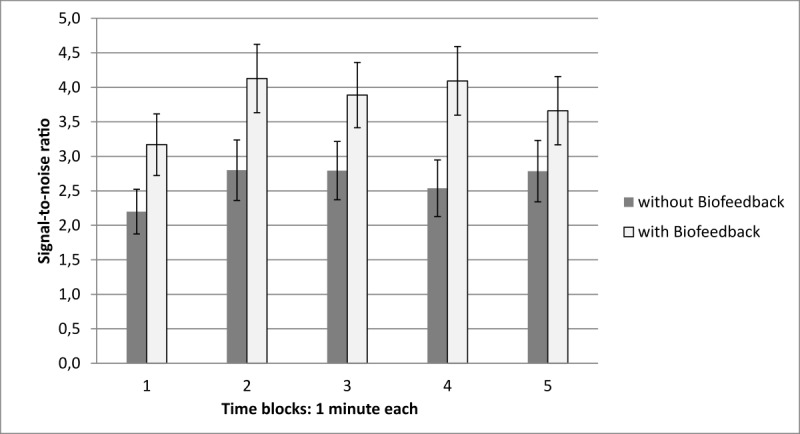
Group comparisons of signal to noise ratio (SNR) in the training block over time. SNR increases after the first minute in both groups. The analysis of variance reveals significant group differences but no interaction with time. Error bars indicate standard errors of the mean.

For the SNR, there was a main effect of time with decreased SNR during the first minute compared with the second minute (CG: *t*_18_=−2.48, *P*=.02; EG: *t*_19_=−2.36, *P*=.03). There was a main effect of group but no interaction between group and time (see Table 6 and Figure 4 for details).

Comparisons for the subjective ratings of the 2 versions of the app are provided in Table 7. There was an overall trend in favor of the app without biofeedback.

**Table 4:** Means and SDs of power of the requested frequency band (0.09-0.11 Hz) and the signal-to-noise ratio in both groups for the 3 measurement blocks.

Statistical value		Power: control group	Power: experimental group	Signal-to-noise ratio: control group	Signal-to-noise ratio: experimental group
**Baseline**					
	Mean	0.0000041	0.0000036	0.31	0.64
	SD	0.0000067	0.0000063	0.57	1.93
**Training**					
	Mean	0.0000234	0.0000277	9.17	12.18
	SD	0.0000243	0.0000171	6.50	12.20
**Post**					
	Mean	0.0000065	0.0000100	0.73	1.28
	SD	0.0000112	0.0000159	1.29	2.59

**Table 5:** Results of the analysis of variance for the power of the recommended frequency in the training block.

Factor	*F* (*df*)	*P* value
Group	0.27 (1,37)	.61
Time	1.30 (4,148)	.27
Group×time	1.01 (4,148)	.41

**Table 6:** Results of the analysis of variance for the signal-to-noise ratio for the recommended frequency in the training block.

Factor	*F* (*df*)	*P* value
Group	4.18 (1,37)	.048
Time	3.75 (4,148)	.006
Group × time	0.78 (4,148)	.54

**Table 7:** Group comparisons of the subjective app ratings [19] with *t* tests for independent samples. Mean, SD, *t* test values, and *P* values are described for each item.

Item (1=strongly disagree, 5=strongly agree)	Mean (SD) CG^a^	Mean (SD) EG^b^	*t* (*df*)	*P* value
The app facilitates relaxation.	3.58 (1.12)	2.90 (1.17)	1.85 (37)	.07
The app is pleasant to use.	4.00 (0.94)	3.70 (0.98)	0.97 (37)	.34
It is easy to follow the app instructions.	4.74 (0.56)	4.15 (1.14)	2.06 (37)	.049
The app effectively teaches how to breathe.	4.37 (1.07)	4.25 (0.91)	0.37 (37)	.71
The app is effective in reducing stress.	3.74 (1.10)	2.90 (1.12)	2.36 (37)	.02
The app is effective in increasing attention to breathing.	4.63 (0.76)	4.55 (0.69)	0.35 (37)	.73

^a^CG: control group.

^b^EG: experimental group.

### Comparison of the Post Measurement Block With the Baseline

To test whether a single 5-min training session is already enough to evoke changes in the abdominal breathing patterns toward the requested breathing frequency (0.1 Hz), we compared the baseline and the postmeasurement block with regard to the spectral power of this frequency in both groups. There were no main effect of measurement block, no effect of group, and no interaction between block and group (see Table 8 for details).

Comparable results were found for the SNR. There was no main effect of measurement block, no main effect of group, and no interaction between group and block (see Table 9 for details, see also Table 4 for mean and SD).

**Table 8:** Results of the analysis of variance for the power of the recommended frequency in the postmeasurement block.

Factor	*F* (*df*)	*P* value
Group	0.49 (1,37)	.49
Block	3.60 (1,37)	.07
Group × block	0.58 (1,37)	.45

**Table 9:** Results of the analysis of variance for the signal-to-noise ratio for the recommended frequency in the postmeasurement block.

Factor	*F* (*df*)	*P* value
Group	1.33 (1,37)	.26
Block	1.60 (1,37)	.21
Group × block	0.07 (1,37)	.80

## Discussion

### Principal Findings

The *Breathing-Mentor* app combines effective visualization of the instruction [19] with biofeedback on deep abdominal breathing, based on the mobile phone’s accelerometers. We conducted a first pilot study with 2 versions of the app to receive the user’s feedback and investigate breathing behavior during the initial 5 min of contact. To reveal possible effects of the biofeedback, both versions contained the same visual instruction, but only the full version included additional biofeedback.

### Effects of the Visual Instruction

The baseline block revealed that both groups were comparable before the breathing training regarding their ability to breathe deeply and slowly with the abdomen. Breathing frequencies near the normal breathing frequency (0.2 Hz) were more prominent in both groups compared with slower frequencies. This shows that participants were rather novices for slow abdominal breathing exercises. This finding agrees with the results from the questionnaire on previous experience with breathing exercises for relaxation purposes. Although most participants were aware that abdominal breathing can be used for relaxation exercises, only few participants actually reported practicing such exercises. Thus, the participants were representative of users who could benefit from a training app for diaphragmatic breathing [19].

Indeed, both versions of Breathing-Mentor (visual instruction only and visual instruction with additional biofeedback) enabled the users to realize the requested breathing frequency of 0.1 Hz more accurately compared with the baseline, as reflected by the spectral power and the SNR. This was expected, as both conditions include the wave-based visual instruction, which has already been shown to be very effective for mobile breathing training [19]. Moreover, SNR increased in both groups after the first minute and remained at a stable level. This fast adaptation of the breathing pattern toward the instructed frequency supports the effectivity of the user interface [19] and goes in line with the high subjective ratings of the ease of use (see Table 7 for details). There is, however, no further improvement within the 5-min training block. Moreover, the postmeasurement block revealed that breathing performance returns to the baseline performance in both groups, when the visual instructions are removed again. Both findings show that the 5-min training block is not enough to trigger transfer learning. Participants remain dependent on the app during the breathing exercise. However, the protection of the users’ autonomy has been identified as an important factor in a recent stress management app [11]. Therefore, additional blocks with terminal feedback without the visual instruction might be 1 possibility to counteract dependency upon the interface and to trigger transfer learning [31].

### Effects of Additional Biofeedback

The main research question of this study was, how additional biofeedback in a mobile app, as implemented in Breathing-Mentor (see the Methods section for details), influences people’s ability to follow the visual breathing instruction and their subjective usage experience.

Although the spectral power of the desired frequency band did not result in significant group differences, the SNR was higher for the biofeedback training group (see the Results section for details). This means that abdominal breathing at the desired frequency was not more prominent compared with the CG without biofeedback, but the occurrence of undesired frequency bands was reduced for the biofeedback group, resulting in enhanced SNR values. These findings support the effectiveness of the additional biofeedback on breathing behavior.

This benefit in performance was, however, combined with lower subjective ratings regarding the effectiveness of the app to reduce stress and ease with which app instructions could be followed for the biofeedback training. This result could be a consequence of increased cognitive workload and attention resources that are required to interpret and modulate the biofeedback graph [32]. Nevertheless, ratings for ease of use and task difficulty were high in both groups. This suggests that workload was not excessive during the training. The physiological stress level and cognitive processing during the training should be addressed more deeply in future studies, as they are expected to change with proficiency level. The role of the relaxation level could be addressed by including additional objective psychophysiological parameters [25-27] to complete the subjective ratings. Cognitive measures could also be targeted with psychophysiological parameters from electroencephalography [33] or eye tracking [34].

### Limitations and Outlook

To summarize, Breathing-Mentor seems to be a useful tool to teach specific abdominal breathing patterns. An immediate improvement of the user’s relaxation state should, however, not be expected, especially for persons who are inexperienced with breathing tasks. With further experience, tools such as the BellyBio Interactive Breathing app might be more useful, as the auditory feedback allows to close the eyes and to focus more intensively on the body, which are facilitating factors for deep relaxation [35]. Such auditory tools might also be useful for people with age-related visual impairments. A multimodal approach could be considered to extend the app to older people.

Finally, the frame that is used to hold the mobile phone at a stable position is 1 limitation factor. Although there were no user complaints concerning the usability of this approach, the correct positioning of the mobile phone was guaranteed by the calibrating procedure and the principal investigator in this study. Other fixing solutions should be considered for everyday use.

### Conclusions

In summary, it should be noted that participants were rapidly able to adjust their breathing pattern to the instruction (within 1 min). This result supports the feasibility and usefulness of biofeedback in mobile breathing apps based on the mobile phone’s accelerometers, especially for people who are unfamiliar with breathing techniques. Immediate effects on the user’s relaxation state should, however, not be expected.

## Abbreviations

CG: control group
EG: experimental group
SNR: signal-to-noise ratio
